# Self-request chest x-ray services—reducing barriers to diagnosis of lung cancer

**DOI:** 10.1093/bjr/tqaf232

**Published:** 2025-09-18

**Authors:** Joanna L Start, Stephen Bradley, Matthew E J Callister, Willy Choon Kon Yune, Tom Daniels, Matthew Evison, Seamus Grundy, Rehan Naseer, Ben Noble, Charlotte A Porter, Bobby S K Bhartia

**Affiliations:** Department of Clinical Radiology, Leeds Teaching Hospitals NHS Trust, Leeds LS9 7TF, United Kingdom; School of Medicine & Population Health, University of Sheffield, Sheffield S10 2TN, United Kingdom; York Street Practice, Leeds LS9 8AA, United Kingdom; Department of Respiratory Medicine, Leeds Teaching Hospitals NHS Trust, Leeds LS9 7TF, United Kingdom; Leeds Institute of Health Sciences, University of Leeds, Leeds LS2 9JT, United Kingdom; Department of Respiratory Medicine, Fairfield General Hospital, Northern Care Alliance NHS Foundation Trust, Bury, Greater Manchester BL9 7TD, United Kingdom; NHS West Yorkshire Integrated Care Board, Wakefield WF1 1LT, United Kingdom; Department of Respiratory Medicine, Wythenshawe Hospital, Manchester University NHS Foundation Trust, Manchester M23 9LT, United Kingdom; Manchester Academic Health & Science Centre, University of Manchester, Manchester M13 9NQ, United Kingdom; Department of Respiratory Medicine, Salford Royal Hospital, Northern Care Alliance NHS Foundation Trust, Salford M6 8HD, United Kingdom; Department of Respiratory Medicine, Fairfield General Hospital, Northern Care Alliance NHS Foundation Trust, Bury, Greater Manchester BL9 7TD, United Kingdom; East Midlands Cancer Alliance, Leicester LE3 8RA, United Kingdom; Woodbrook Medical Centre, Loughborough LE11 1NH, United Kingdom; Department of Radiological Physics, Leeds Teaching Hospitals NHS Trust, Leeds LS9 7TF, United Kingdom; Department of Clinical Radiology, Leeds Teaching Hospitals NHS Trust, Leeds LS9 7TF, United Kingdom

**Keywords:** lung cancer, chest x-ray, diagnosis, self-request, self-referral, general practice, social deprivation, barriers

## Abstract

Despite the welcome introduction of the NHS Lung Cancer Screening Programme, most diagnoses of lung cancer will continue to be made via symptomatic and emergency presentations. Multiple patient-related factors act as a barrier to symptomatic presentation and eventual diagnosis including perceptions of personal risk and a view that the often non-specific symptoms may be unworthy of medical attention in an ever-busy NHS. Self-request chest x-ray services rolled out in Leeds and Greater Manchester aim to reduce these barriers to diagnosis in order to achieve earlier diagnosis of lung cancer and improve outcomes. The services have been accessed over 14 000 times. Lung cancer is a leading cause of health inequality, and these services effectively target populations with high levels of deprivation who are at the greatest risk of lung cancer and yet face the greatest barriers to diagnosis. Self-request services empower patients to take ownership of their health. Supported by organizations representing those affected by lung cancer, the initiative is a step towards restoring access to healthcare. This commentary discusses the rationale for the self-request chest x-ray service and describes the service model, addressing areas of potential concern and controversy.

## The challenge

Healthcare professionals supported by the Roy Castle Lung Cancer Foundation are calling for people with symptoms of potential lung cancer to be able to self-request a chest x-ray (CXR) rather than requiring x-ray referral from a doctor.^1^ The NHS supports policies which empower patients and streamline access to relevant services.^2^ This commentary outlines the rationale for delivering such a service, how it may help achieve earlier diagnosis of lung cancer and addresses areas of concern such as increased workload in primary care and radiology departments.

Lung cancer is the leading cause of cancer death in the UK and a major cause of health inequality; those living in England’s most deprived areas are twice as likely to die from lung cancer compared with those in the least deprived.^3^ Late presentation resulting in a more advanced stage at diagnosis is thought to be a significant contributing factor, with 45% of individuals diagnosed with lung cancer in England presenting with stage IV disease and 32% diagnosed via an emergency presentation.^4^

The NHS Lung Cancer Screening Programme is a welcome step towards addressing this issue. Those eligible are identified using risk prediction models from people aged 55-74 years with a history of smoking, and screening is offered with low-dose CT. However, less than half of individuals with lung cancer meet current eligibility requirements,^5^ and currently only about half of those invited choose to participate.^6^ Symptomatic presentations will therefore continue to be the route to diagnosis for most patients with lung cancer.

The National Institute for Health and Care Excellence (NICE) provides guidance for the investigation of symptomatic individuals based on age, smoking status and the presence of specific signs and symptoms. The guidelines recommend CXR as the initial investigation for the most common symptoms of possible lung malignancy, with urgent referral for further investigation recommended if the CXR demonstrates abnormalities suggestive of lung cancer.

The indications endorsed by NICE for performing a CXR have low positive predictive power and set a low threshold for performing the examination. Liberal use of the investigation is also associated with better outcomes. A recent analysis of CXR investigation rates amongst 7400 primary care practices in England demonstrated that patients from practices with the highest rates of investigation had fewer advanced stage lung cancers (OR 0.87; 95% CI, 0.83-0.92) and a significantly reduced risk of death within 1 and 5 years of diagnosis (HR 0.92; 95% CI, 0.90-0.95 and HR 0.95; 95% CI, 0.91-0.99, respectively).^7^ A lung cancer symptom awareness campaign in Leeds in 2011 led to an 81% increase in community-requested CXR and was associated with a 9% reduction in the number of patients diagnosed with stage III and IV lung cancer.^8^

The greatest delays in lung cancer diagnostic pathways are thought to occur between the initial development of symptoms and seeking medical help, described as the “within-patient” delay. A study of 379 patients diagnosed with lung cancer identified that this initial delay contributed to over half of the pathway time between developing symptoms and beginning treatment.^9^ The reasons are complex and include awareness of potential symptoms of cancer, perception of personal risk, the stigma related to smoking, anxiety, and the barriers to accessing healthcare, both real and perceived. In international comparisons of public attitudes, the UK public perceived the greatest barriers in obtaining medical evaluations.^10^ The reasons cited for not seeking help with symptoms of potential lung cancer included personal embarrassment and, in one third of respondents, concerns about wasting their doctor’s time. Many fear that symptoms would be dismissed by a doctor or are not worthy of consulting an ever-busy NHS, especially after the COVID-19 pandemic.^11^

Once evaluated, patients also face difficulties in accessing a CXR despite it being a safe, relatively cheap, and widely available test. Both the patient and clinician may struggle to recognise the significance of often non-specific symptoms, especially given the relative infrequency of lung cancer in a primary care setting. Cough is the most frequent symptom of lung cancer, yet fewer than 1 in 200 of those presenting with a cough to primary care will subsequently be diagnosed with lung cancer.^12^ In a recent national evaluation of 162 000 primary care consultations, less than 1 in 7 patients with newly presenting dyspnoea were offered an urgent CXR as advised.^13^ Individuals with a history of smoking, those from socially deprived backgrounds or with co-morbid conditions were less likely to be offered prompt imaging and more likely to be diagnosed via an emergency presentation.

## The self-request chest x-ray service model

The self-request CXR service addresses these barriers by allowing individuals to undergo a CXR without the need to seek the consent of a clinician. The concept was first tested in Leeds in 2011 and subsequently piloted in Corby, Northumbria and Greater Manchester. Further services are being piloted in Leicestershire and within Northern Cancer Alliance.

**Figure 1.**
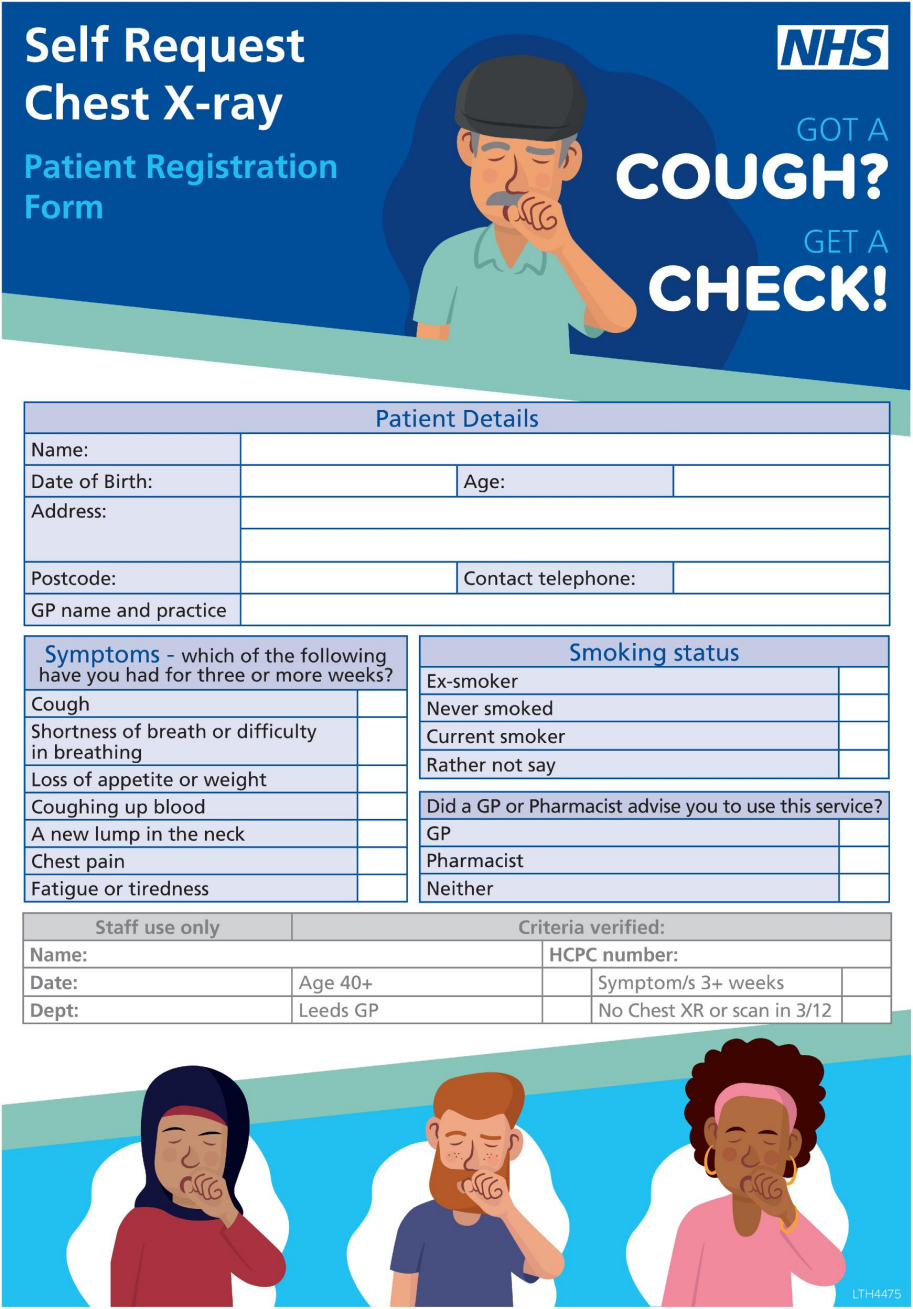
Patient registration form for the self-request chest x-ray service.

The models for the different services are similar in design (Table 1). The services are open to adults aged 40 years or over who are registered with a local general practitioner (GP), who present with at least one symptom described by NICE as an indication for investigation with CXR or with haemoptysis (Figure 1). Access is on a walk-in basis to participating radiology departments. The patient completes a written form, and the radiographer confirms the patient’s eligibility using a local agreed protocol and performs the CXR.

**Table 1.** Overview of self-request CXR services in Leeds and Greater Manchester.

	Leeds	Greater Manchester
Eligibility	Age ≥40 with defined symptom as per patient registration form for ≥3 weeks and no chest imaging in last 3 months	
Geographic availability (population)	Throughout Leeds, with promotion focused on high-deprivation PCNs (∼870 000)	Patients registered with GP in 2 localities: Bury and “Heywood, Middleton & Rochdale” (∼450 000)
Service website	www.leedsth.nhs.uk/gotacough	https://gmcancer.org.uk/chestxray/
Access to service	Walk-in	
IR(MER) referrer	Radiographer	
Named responsible clinician	GP	Respiratory physician
Provider	Leeds Teaching Hospitals NHS Trust	Northern Care Alliance NHS Foundation Trust
Key partners	West Yorkshire ICB & Cancer Alliance, Leeds City Council Public Health team	Greater Manchester Cancer Alliance
Service initiated	2011 (paused 2019-2021)	2022
Number of chest x-rays performed (evaluation period)	8948 (2011-2016)	3751 (July 2022-July 2024)
Percentage accessing who are current or ex smokers	67%	53%
Lung cancer yield during the evaluation period (yield including other types of intrathoracic cancers)	0.9% (1.1%)	0.5% (0.7%)

To comply with Ionising Radiation (Medical Exposure) Regulations (IR(ME)R), the referrer must be a registered healthcare professional who has been appointed by the employer. A secondary care physician, the patient’s GP and the radiographer who assesses the patient have all been used as the referrer in the different models. The responsible clinician to whom the final report is directed may be the patient’s GP or a secondary care physician. The Care Quality Commission have advised that each service requires its own local Medical Physics approval and to ensure there are clear written protocols with appropriate audit of the service.

Written safety netting advice is provided to the patient regarding the necessity of obtaining the results of the investigation. Given the limited sensitivity of the CXR for the detection of lung cancer^14^ and to avoid false reassurance from a normal result, the patient is provided guidance to seek further assistance if symptoms persist or worsen.

**Figure 2.**
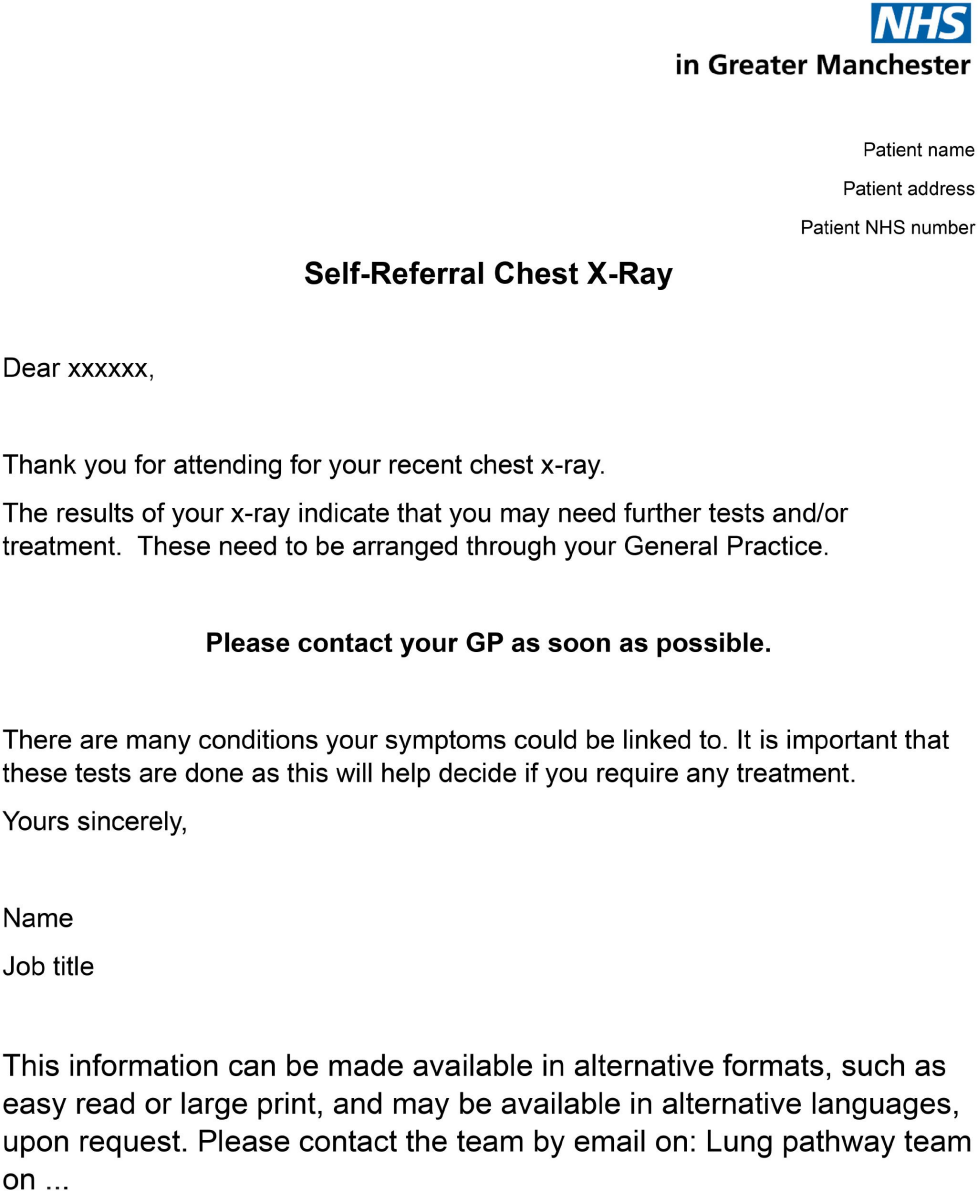
Standard letter to patient if self-request chest x-ray (CXR) abnormal.

As a GP may be unaware that the patient has attended, the verified report includes a statement to the GP indicating that the patient has self-requested the CXR, and the GP is alerted that the investigation has taken place. Results are communicated either to the GP whom the patient is instructed to consult for the result, or by writing to the patient directly using a standard proforma letter (Figure 2). Patients can also access their results via the NHS App, and the different services are evaluating patient feedback to optimize these processes.

## Are the services used appropriately?

The longest established services in Leeds and Greater Manchester have been accessed more than 14 000 times. Patient feedback indicates that convenience and reassurance provided by the service were the commonest reasons given for approaching the services. Service evaluation of user demographics indicates a mean age of 62 years, of whom 51% either currently smoke or have previously smoked. 38% attended from areas within the highest quintile of social deprivation with the highest risks of lung cancer, highlighting that the service is being accessed by those at greatest risk and not the “worried well.” There were 16 different first languages spoken across the cohort.^15^

**Figure 3.**
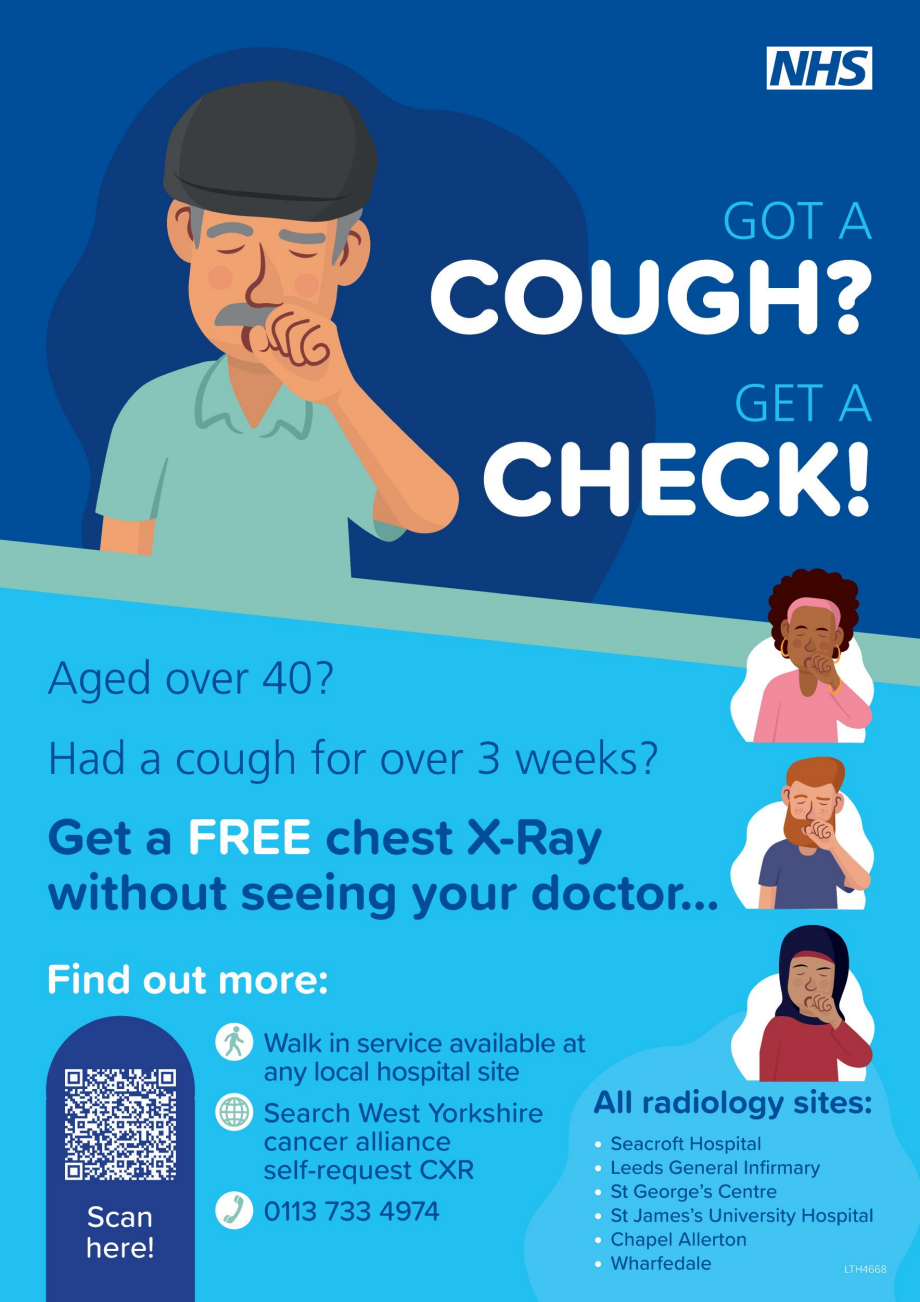
Poster advertising the self-request chest x-ray service in Leeds.

**Figure 4.**
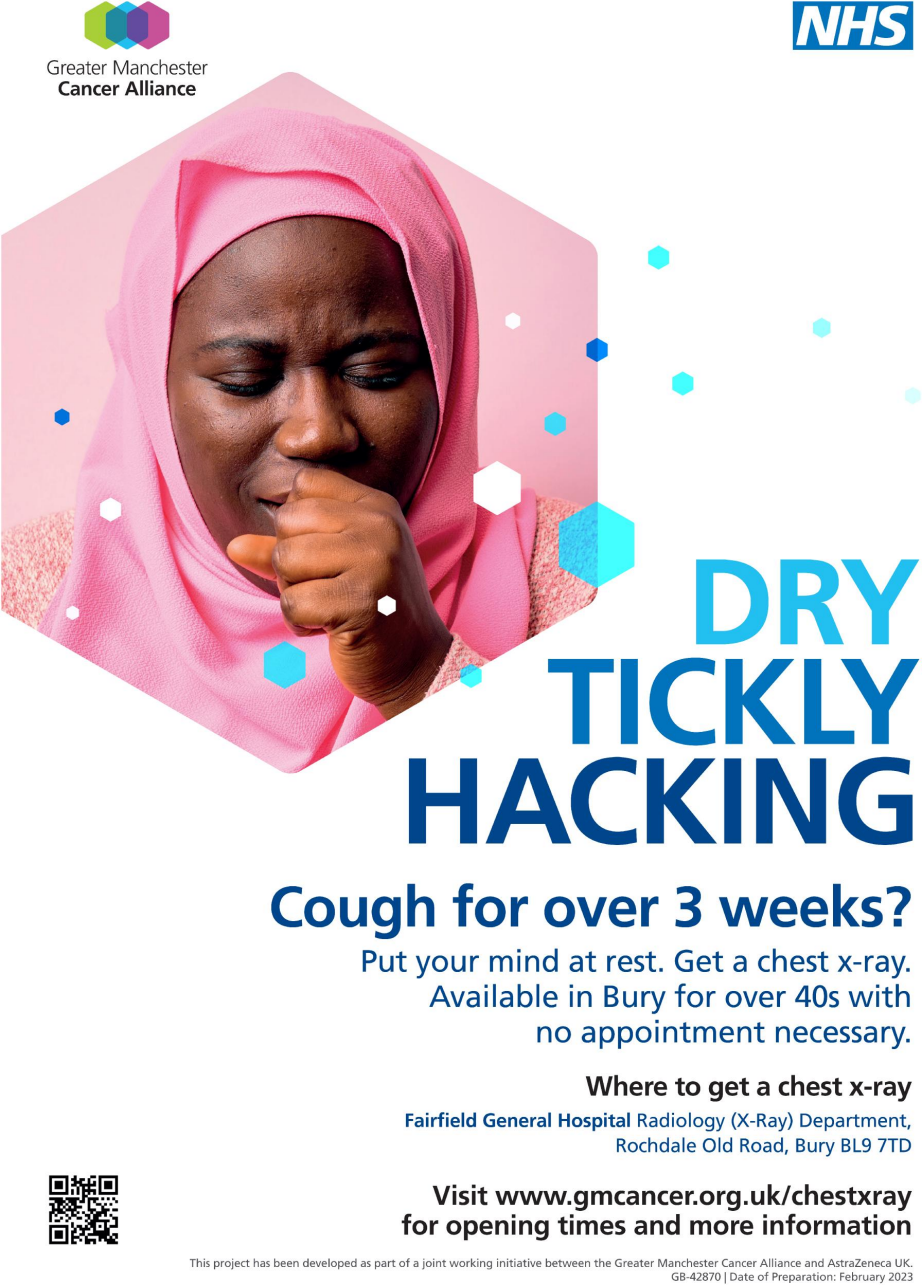
Poster advertising the self-request chest x-ray service in Greater Manchester.

In order to try to achieve equity of access, there has been promotion of the service via local public health teams, the press and social media, and using a variety of languages including Bengali and Urdu. A variety of different posters are used across the Leeds and Manchester services (Figures 3 and 4, respectively). Integration into standard clinical pathways such as the NHS 111 service is currently being evaluated.

Approximately 1% of users are diagnosed with lung cancer because of their self-request CXR. Among all of those attending the underlying probability of developing lung cancer within 2 years of a CXR is 1.5%, which is comparable to the national figure of 1.4% amongst GP referral CXRs.^16^ How lung cancer outcomes compare to standard pathways is the subject of current evaluation and will be published in due course.

## Workload implications

The most longstanding self-request service in Leeds has led to an increase in the number of CXRs performed in those aged over 40 referred from primary care. Self-request CXRs made up 5% of the total number of community-ordered CXRs between 2011 and 2015.^8^ The majority of CXRs are normal or demonstrate benign abnormalities requiring no further follow up. Up to 7% of CXRs may reveal findings which require further clinical assessment or follow up imaging organized by the GP.^14^ Although this may be viewed as additional work for the GP, many patients avoid an initial assessment visit prior to the test. For findings suggestive of lung cancer, individuals are automatically recalled for a CT scan via an established electronic pathway, negating the requirement for the GP to request the scan. These cases are discussed within the multidisciplinary team meeting, standardizing care and minimizing overdiagnosis.

## Conclusions

Most patients with lung cancer will be diagnosed through symptomatic pathways, with the CXR remaining the key initial test. Barriers which prevent access to this investigation contribute to delays in diagnosis and poorer outcomes. The self-request CXR service empowers patients and is one means of addressing these barriers. The concept is supported by organizations representing those affected by lung cancer and shows promising results across different parts of England, successfully targeting communities with the highest levels of deprivation at the highest risk of lung cancer.
